# Complete genome sequences of *Mycoplasma cynos* and *Mycoplasma felis* isolated from dogs and cats with infectious respiratory disease

**DOI:** 10.1128/mra.01243-23

**Published:** 2024-03-21

**Authors:** Isaac Framst, Priyatharshini Ramesh, Hugh Y. Cai, Grazieli Maboni

**Affiliations:** 1 Department of Pathobiology, Ontario Veterinary College, University of Guelph, Guelph, Ontario, Canada; 2 Animal Health Laboratory, University of Guelph, Guelph, Ontario, Canada; 3 Department of Infectious Diseases, Athens Veterinary Diagnostic Laboratory, College of Veterinary Medicine, University of Georgia, Athens, Georgia, USA; University of Maryland School of Medicine, Baltimore, Maryland, USA

**Keywords:** *Mycoplasma cynos*, *Mycoplasma felis*, nanopore, Illumina, hybrid assembly

## Abstract

*Mycoplasma cynos* is a primary agent of pneumonia in dogs, and *Mycoplasma felis* is associated with upper respiratory tract disease in cats. We present complete genome sequences of 26 isolates from clinically affected dogs and cats. These genome sequences will facilitate new molecular and epidemiological analyses.

## ANNOUNCEMENT

*Mycoplasma cynos* and *Mycoplasma felis* are fastidious respiratory bacteria of dogs and cats, respectively. *Mycoplasmopsis* is now considered a homotypic synonym of the genus *Mycoplasma*; however, this term is not widely used yet. *Mycoplasma cynos* is a primary pathogen causing fatal pneumonia in dogs (1–3). *M. cynos* is also associated with canine infectious respiratory disease complex; however, its pathogenicity and role in co-infections with other bacteria and viruses are not well understood. *Mycoplasma felis* is a pathogen of cats mostly associated with feline upper respiratory tract disease and conjunctivitis (4, 5). Similarly, little is known about its pathogenicity, occurrence, and antimicrobial susceptibility profile. Treatment of *Mycoplasma*-associated respiratory disease in dogs and cats is based primarily on the empirical use of tetracyclines (6). Whole genome sequences can provide insight on the virulence, antimicrobial susceptibility, comparative genomics, and phylogenetic relationship of these pathogens. Currently, the availability of whole genome sequences of *M. cynos* and *M. felis* in public databases is limited.

*M. cynos* (*n* = 11) and *M. felis* (*n* = 15) archived isolates were obtained from the Animal Health Laboratory (Ontario Veterinary College, University of Guelph, Canada). Bacterial isolates were cultured using a modified Hayflick’s broth for 96 hours at 37°C and 5% CO_2_ (7). DNA extraction was performed using the GenElute Bacterial Genomic DNA Extraction Kit (Sigma-Aldrich) (7). For each isolate, two sequencing platforms, Illumina MiSeq and Oxford Nanopore Technologies (ONT) minION, were used. Libraries for ONT sequencing were prepared with the rapid barcoding kit (SQK-RBK110.96) and sequenced using R9.4.1 flow cell. The Nextera DNA Flex Kit was used to prepare DNA library for Illumina sequencing (Advanced Analysis Centre, University of Guelph).

For bioinformatic analysis, default parameters were used unless indicated otherwise. Nanopore reads were basecalled, trimmed, and demultiplexed using Guppy v6.2.1 (high-accuracy model) and Porechop V0.2.4 (https://github.com/rrwick/Porechop). Pair-end Illumina reads were trimmed merged by sequence name using BBduk v39.00 and BBmerge v39.00 with maq = 10 quality filtering (https://jgi.doe.gov/data-and-tools/software-tools/bbtools). The read length was 301 base pairs for all Illumina sequences. Using both ONT and Illumina reads, hybrid assemblies were generated using hybrid SPAdes v3.15.5 (https://github.com/ablab/spades) and polished using Pilon v1.24 (https://github.com/broadinstitute/pilon) (8, 9). Genomes were circularized using MAUVE (https://darlinglab.org/mauve/mauve.html). Assembly quality and completeness were assessed using QUAST v5.2.0 (https://github.com/ablab/quast) and BUSCO v.5.5.0 (https://busco.ezlab.org) (10, 11).

**TABLE 1:** Metrics and accession numbers of genome sequences of *Mycoplasma cynos* and *Mycoplasma felis* isolates from dogs and cats

Sample ID	Species	Genome size (bp)	GC content (%)	Illumina data				Oxford Nanopore data			SRA accession no.		
				No. reads	Average read length (bp)	Coverage (x)		No. reads	N50 (bp)	Coverage (x)	ONT	Illumina	Genome assembly
C01	*M. cynos*	949,492	25.9	133,035	301	42		65,458	11,298	349	SRR26841336	SRR27086663	CP141046
C02	*M. cynos*	944,354	25.73	190,639	301	61		42,185	10,302	221	SRR26841316	SRR27086662	CP141045
C03	*M. cynos*	1,008,822	25.75	391,642	301	117		46,913	12,830	314	SRR26841315	SRR27086651	CP141044
C04	*M. cynos*	988,379	25.66	656,058	301	200		36,387	2,443	32	SRR26841314	SRR27086645	CP141043
C06	*M. cynos*	1,001,136	25.74	271,942	301	82		232,207	2,941	268	SRR26841313	SRR27086644	CP141042
C07	*M. cynos*	1,001,942	25.55	242,115	301	722		209,498	1,384	432	SRR27145255	SRR27145254	CP140753
C09	*M. cynos*	1,480,121	26.09	803,38	301	16		92,536	6,811	184	SRR26841312	SRR27086643	CP141041
C10	*M. cynos*	978,596	25.66	342,071	301	105		25,497	11,367	56	SRR26841335	SRR27086642	CP141040
C11	*M. cynos*	1,011,799	25.66	323,920	301	96		182,671	7,958	368	SRR26841324	SRR27086641	CP141039
C12	*M. cynos*	985,438	25.74	822,806	301	251		275,784	4,507	915	SRR26841318	SRR27086640	CP141038
C14	*M. cynos*	1,008,656	25.69	210,244	301	63		216,351	4,990	487	SRR26841317	SRR27086639	CP141036
F01	*M. felis*	1,009,149	24.37	530,188	301	158		225,051	5,359	422	SRR26841334	SRR27086661	CP141035
F02	*M. felis*	934,836	24.34	311,460	301	100		178,772	4,272	309	SRR26841326	SRR27086660	CP141034
F03	*M. felis*	964,027	24.49	437,769	301	137		217,547	6,161	495	SRR26841325	SRR27086659	CP141033
F04	*M. felis*	987,588	24.47	390,691	301	119		54,316	5,955	157	SRR26841323	SRR27086658	CP141032
F06	*M. felis*	947,125	24.63	425,027	301	135		150,158	2,554	261	SRR26841322	SRR27086657	CP141031
F07	*M. felis*	919,762	24.52	311,572	301	102		218,664	3,428	342	SRR26841321	SRR27086656	CP141030
F08	*M. felis*	958,349	24.46	309,239	301	97		189,203	3,512	293	SRR26841320	SRR27086655	CP141029
F09	*M. felis*	871,729	24.42	227,247	301	78		69,437	2,476	99	SRR26841319	SRR27086654	CP141028
F10	*M. felis*	955,761	24.48	342,085	301	108		95,271	3,081	144	SRR26841333	SRR27086653	CP141047
F11	*M. felis*	926,561	24.7	441,056	301	143		293,087	2,554	383	SRR26841332	SRR27086652	CP141027
F12	*M. felis*	890,049	24.55	585,746	301	198		338,849	3,083	496	SRR26841331	SRR27086650	CP141026
F13	*M. felis*	869,853	24.44	998,956	301	346		33,200	2,629	52	SRR26841330	SRR27086649	CP141025
F14	*M. felis*	948,290	25.39	256,905	301	82		60,651	2,132	68	SRR26841329	SRR27086648	CP141024
F15	*M. felis*	944,886	24.27	108,964	301	35		448,368	2,101	601	SRR26841328	SRR27086647	CP141023
F16	*M. felis*	953,413	24.7	387,787	301	122		106,943	2,333	124	SRR26841327	SRR27086646	CP141036

## Data Availability

The data were deposited to the NCBI GenBank database under the BioProject accession no. PRJNA1018939. The complete sequences and their corresponding raw reads have been deposited in the GenBank and the Sequence Read Archive (SRA) (Table 1).
